# Solitary Encapsulated Neurofibroma Not Associated with Neurofibromatosis-1 Affecting Tongue in a 73-Year-Old Female

**DOI:** 10.1155/2016/3630153

**Published:** 2016-07-20

**Authors:** Sk. Abdul Mahmud, Neha Shah, Moumita Chattaraj, Swagata Gayen

**Affiliations:** Department of Oral & Maxillofacial Pathology, Guru Nanak Institute of Dental Sciences & Research, 157/F Nilgunj Road, Panihati, Kolkata 700114, India

## Abstract

Neurofibromas are benign tumors of nerve cell origin arising due to proliferation of Schwann cells and fibroblasts. They are usually asymptomatic and hence remain undiagnosed. They are commonly found on the skin and intraorally tongue is the most common site for their occurrence. Here, we present a unique case of solitary encapsulated neurofibroma in the oral cavity without any clinical manifestations or family history of Neurofibromatosis type 1 in a 73-year-old female patient who presented with a painless swelling on the tongue. The histopathologic findings closely mimicked benign fibrous histiocytoma. In our case, definitive diagnosis of neurofibroma was made based on clinical findings, family history, and histopathological and immunohistochemical evaluation. Through this case report we want to emphasize the role of biopsy and immunohistochemistry in arriving at a confirmatory diagnosis. The patient was treated by surgical excision and showed no signs of recurrence over a follow-up period of 12 months.

## 1. Introduction

Tongue is a complex organ comprising different types of tissues. As such, it can harbor many pathological entities from hamartomas to neoplasms of varied origin. One such neoplasm occurring in the tongue is neurofibroma which is a benign peripheral nerve sheath tumor arising due to proliferation of Schwann cells, perineurial cells, and endoneurial fibroblasts [1–5]. It can present either as a localized lesion or as part of generalized syndrome known as Neurofibromatosis type 1 (NF-1) or Von Recklinghausen's disease or rarely with Type III Multiple Endocrine Neoplasia (MEN-III) Syndrome [1–4, 6]. Based on clinical presentation, it is classified as solitary or multiple [1–6]. Intraorally, it can be further classified as “intraosseous” and “extraosseous” [7].

Solitary neurofibroma is commonly found in skin [3, 6]. It is rarely seen in the oral cavity [1, 3]. Intraorally, only 6.5% of cases of solitary neurofibromas have been reported which are not associated with NF-1 [4]. It can affect a wide age group from 10 months to 70 years, though it is commonly found in 3rd decade [3, 6]. The most common intraoral site is tongue followed by buccal mucosa, floor of the mouth, palate, lips, and gingiva [3–8].

Clinically, it presents as submucosal, slow growing, soft, sessile usually painless lesion that may vary in size from small nodule to large mass. The overlying mucosa of solitary neurofibroma not associated with NF-1 gradually blends with the surrounding normal mucosa and there is no clear-cut demarcation between the lesion and normal mucosa [3, 9].

Histologically, solitary neurofibroma is usually unencapsulated and consists of interlacing bundles of spindle shaped cells with wavy nuclei in fibrous or myxomatous stroma [1, 2, 5, 9]. Numerous mast cells and lymphocytes are usually scattered in the connective tissue [1, 2, 4–6, 8–10].

Immunohistochemically, the tumor cells show positive reactivity for S-100 protein (in 85% to 100% of cases), neuron specific enolase, and vimentin [1, 2, 7, 8]. Complete surgical excision is the treatment of choice [2–4, 9, 10]. The rate of malignant transformation is about 3% to 15%, especially when associated with NF-1 [3, 4, 10].

Here, we present a unique and rare case of solitary encapsulated neurofibroma not associated with NF-1 in the tongue in a 73-year-old female patient.

## 2.  Case Report

A 73-year-old female patient reported in the Department of Oral & Maxillofacial Pathology of Guru Nanak Institute of Dental Sciences and Research, Kolkata, India, with the chief complaint of a painless swelling present over the tongue for the last four months. The swelling had increased gradually to attain the present dimension. The patient did not complain of any difficulty in swallowing, chewing, tongue movement, speech, and breathing. She had no deleterious oral habit. Her medical history revealed hyperthyroidism which was controlled with medication. General physical examination showed a moderately built, nourished female with steady gait and satisfactory vital signs. There were no signs of clubbing, anaemia, and cyanosis. Extraoral examination revealed nothing significant. Cervical lymph nodes were not palpable.

**Figure 1:**
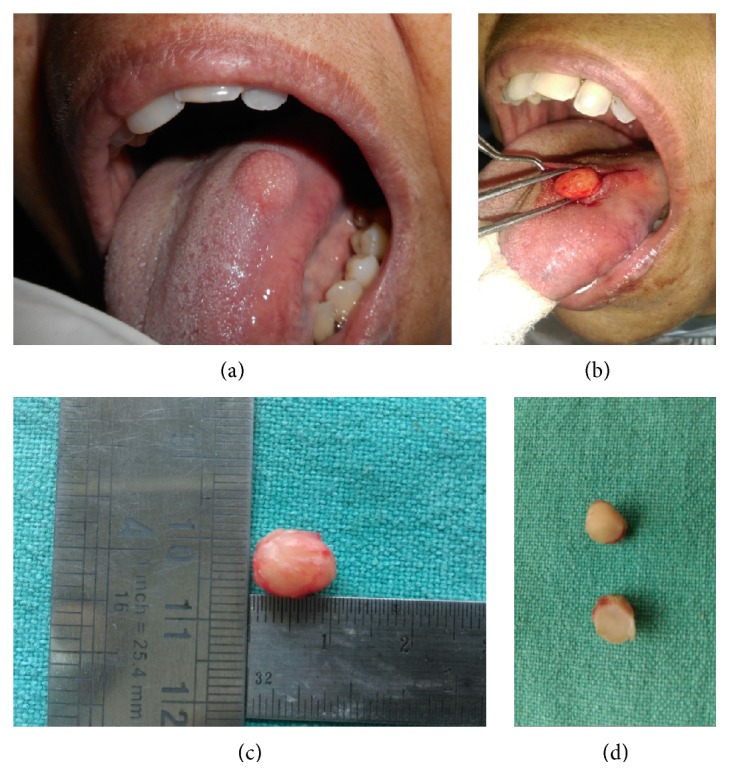
(a) Intraoral photograph showing well-circumscribed round 1.0 cm × 1.0 cm nodule over dorsum of tongue near left lateral border. (b) Peroperative photograph showing well-circumscribed tumor separated from the surroundings through blunt dissection. (c) Gross specimen appearing well-circumscribed, yellowish white, and oval and measuring 10.0 mm × 5.0 mm × 5.0 mm. (d) Cut surface of the excised tumor appearing homogenous, white, and firm without haemorrhage or necrosis.

Intraoral examination revealed the presence of a well-circumscribed, pale pink, round, single, soft to firm, sessile, nontender, nonpulsatile, slightly mobile nodule measuring about 1.0 cm × 1.0 cm over the dorsum of tongue near the left lateral border. The overlying mucosa was nonulcerated and without any vascular prominence (Figure 1(a)). Oral hygiene of the patient was good. There was presence of carious broken left lower first molar and sharp cuspal edges of left upper first molar.

Based on the clinical examination and history given by the patient, the growth was thought to be a benign neoplasm and a provisional diagnosis of “fibroma” was made. Our differential diagnosis included lipoma, traumatic neuroma, neurofibroma, schwannoma, fibrous histiocytoma, granular cell tumor, leiomyoma, and rhabdomyoma.

The patient's routine haemogram was found to be within normal limits. After written informed consent from the patient, an excisional biopsy was performed under local anaesthesia. A vertical incision was given to expose the tumor mass from the overlying mucosa and it was gradually separated from the surroundings through blunt dissection (Figure 1(b)).

The gross specimen was well-circumscribed, yellowish white, and oval shaped and measured 10.0 mm × 5.0 mm × 5.0 mm in dimension (Figure 1(c)). Cut surface was homogenous, white, firm, and without any evidence of haemorrhage or necrosis (Figure 1(d)).

**Figure 2:**
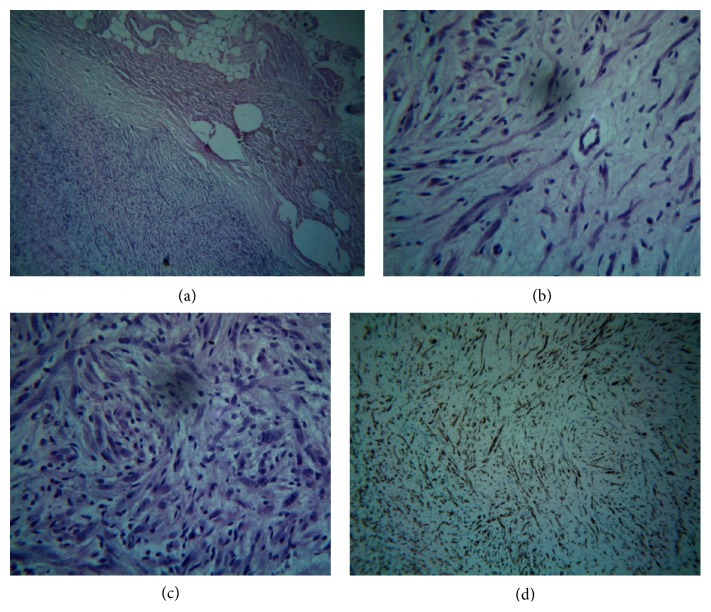
(a) Low power photomicrograph (H&E, 10x) showing fibrous capsule, surrounding the tumor mass. (b) High power photomicrograph (H&E, 40x) showing tumor mass consisting of fibrovascular, myxoid connective tissue stroma interspersed with elongated spindle shaped cells having wavy nuclei. (c) High power photomicrograph (H&E, 40x) showing multiple proliferating spindle shaped and round cells with pale round to oval nuclei. (d) High power photomicrograph (immunoperoxidase staining, 40x) showing strong immunoreactivity for S-100.

Histopathological examination of the section stained with haematoxylin & eosin revealed the presence of a fibrous capsule at some places (Figure 2(a)). The tumor consisted of myxoid connective tissue stroma interspersed with numerous vascular spaces and elongated spindle shaped cells having wavy nuclei (Figure 2(b)). There was also proliferation of multiple spindle shaped cells and round cells with pale round to oval nuclei (Figure 2(c)). Based on the histopathological evaluation, a differential diagnosis of “neurofibroma” and “benign fibrous histiocytoma” was considered. Further, toluidine blue staining of the soft tissue section revealed numerous mast cells scattered in the fibrous stroma. Immunohistochemistry using vimentin, S-100, and CD-68 was done for confirmatory diagnosis. The tumor showed strong immunoreactivity for vimentin and S-100 and negative immunoreactivity for CD-68 (Figure 2(d)). Based on the clinical, histological, and immunohistochemical findings, a definitive diagnosis of “neurofibroma” was made.

The patient was further examined and her family history was elicited. Absence of café-au-lait spots, Lisch nodules, and axillary freckling and no history of similar findings or growth in the family members helped to rule out NF-1. There were no signs of recurrence or NF-1 over a follow-up period of 12 months.

## 3. Discussion

The lesions on tongue pose a diagnostic challenge, because clinically they mimic variety of neoplasms occurring in that region which present with similar clinical features. Neurofibroma is one such neoplasm, commonly found intraorally in the tongue. It is a benign tumor of neural cell origin and characterized by proliferation of Schwann cells and perineurial fibroblasts [1–5]. Shklar and Meyer classified neurofibroma as solitary and multiple [7]. Multiple neurofibromas are generally present as part of generalized syndrome of Neurofibromatosis type 1 or Type III Multiple Endocrine Neoplasia Syndrome [1–4, 6]. Solitary intraoral neurofibroma not associated with NF-I is very rare in the oral cavity [1, 3]. It was first described by Bruce in 1954 [6].

The pathogenesis of solitary neurofibroma not associated with NF-I is poorly understood. Storlazzi et al. using G-banding and fluorescence in situ hybridization analysis showed that somatic inactivation of NF-1 gene located on chromosome 17 through chromosomal translocation leads to increased and abnormal production of neurofibromin which regulates ras-mediated cell growth pathway leading to increased levels of activating proteins p21^ras^ and p13 which causes cellular proliferation of Schwann cells associated with neurofibroma [11].

Neurofibroma has been reported in a wide age range from 10 months to 70 years, in our case the patient was 73 years old, and this deserves a special mention, since solitary neurofibroma predominantly affects younger individuals in the 3rd decade [3, 4, 6]. Our patient presented with a swelling on the tongue which is in accordance with literature as that is the most common intraoral site for occurrence of neurofibroma [3–8].

Clinically, when the tumor on tongue is large in size it can cause difficulty in swallowing, mastication, chewing and respiratory obstruction. Impingement on nerve may lead to pain and paresthesia [9]. No such complaints were noted in this case. Intraoral lesions of neural tissues mainly originate from the branches of fifth, seventh, and rarely ninth cranial nerves. In our case, although it involved the anterior two-thirds of tongue, since there was no alteration of general or special sense hence the cranial nerve branches from which the tumor may have arisen remained unclear [6].

Histologically, most neurofibromas are unencapsulated tumors and consist of elongated fibroblasts with bent, wavy, and serpentine nuclei separated by abundant fine collagen fibres [1–3, 6, 9]. Mast cells are typically found and contribute to fibroblastic proliferation and growth of neurofibroma [12, 13]. In our case we found capsule in some areas along with presence of numerous mast cells scattered in fibrovascular, myxoid connective tissue stroma. Bleeding may be encountered at the time of surgery due to the presence of numerous vascular spaces [8]. The presence of capsule further highlights the unusual nature of the case, since encapsulation is seen in only 4% of neurofibromas [14]. Further, this case is unique as the presentation of the lesion was sporadic without any associated family history.

Solitary neurofibromas may become malignant, although it is extremely rare. Malignant transformation is usually seen with multiple neurofibromas and those associated with Von Recklinghausen disease or MEN-III syndrome [3, 4, 10]. Though recurrence of solitary neurofibroma is rare but it has been reported that there is a higher rate of recurrence in the head and neck region. Also, the tumors which have been excised several times may turn malignant [5].

The patient was treated by complete excision of the tumor which was in accordance with the treatment protocol for solitary neurofibroma [2–4, 9, 10]. No signs of recurrence or NF-1 were noted over a follow-up period of 12 months.

## 4. Conclusion

The lesions on the tongue pose a diagnostic challenge as clinically they can mimic a variety of neoplasms. Biopsy followed by histopathological evaluation remains the gold standard for arriving at a diagnosis. In our case as the lesion had histopathologic similarity with other lesions, hence immunohistochemistry was pivotal for confirmatory diagnosis of neurofibroma.

Occasionally, solitary neurofibroma may be the first manifestation of the generalized syndrome of Neurofibromatosis type 1. Hence, periodic follow-up of the patient is very essential since recurrence and malignant changes have been reported in such cases.
